# Long non-coding RNA FOXP4-AS1 is a prognostic biomarker and associated with immune infiltrates in ovarian serous cystadenocarcinoma

**DOI:** 10.1097/MD.0000000000027473

**Published:** 2021-10-08

**Authors:** Cheng Liao, Ao Wang, Yushan Ma, Hui Liu

**Affiliations:** a Department of Anesthesiology, West China Second University Hospital, Sichuan University, Key Laboratory of Birth Defects and Related Diseases of Women and Children, Sichuan University, Ministry of Education, Chengdu, Sichuan, P. R. China; b Department of Gynecology and Obstetrics, West China Second University Hospital, Sichuan University, Key Laboratory of Birth Defects and Related Diseases of Women and Children, Sichuan University, Ministry of Education, Chengdu, Sichuan, P. R. China.

**Keywords:** biomarker, FOXP4-AS1, ovarian serous cystadenocarcinoma, prognosis, The Cancer Genome Atlas

## Abstract

**Background:**

FOXP4-AS1 expression participates in multiple signal pathways and has been previously reported in colorectal cancer, cervical cancer, and other cancer cells. However, its role on prognosis and immune infiltrates in ovarian serous cystadenocarcinoma (OVs) remains unclear. The purpose of our study was to investigate the expression of FOXP4-AS1 in OVs and its association with immune infiltrates, and determined its prognostic roles in OVs.

**Methods:**

Using The Cancer Genome Atlas (TCGA) database, we retrieved FOXP4-AS1 expression and clinical information for 376 patients with OVs. Wilcoxon rank sum test was used to compare the expression of FOXP4-AS1 in OVs and normal ovarian tissue. Logistic regression was used to analyze the relationship between clinicopathologic features and FOXP4-AS1. Gene Set Enrichment Analysis (GSEA), and single sample Gene Set Enrichment Analysis (ssGSEA) was conducted to investigate the enrich pathways and functions and quantify the extent of immune cells infiltration for FOXP4-AS1. Kaplan–Meier method was used to generate survival curves, and Cox regression was used to analyze the relationship between FOXP4-AS1 and survival rate.

**Results:**

High FOXP4-AS1 expression was significantly correlated with tumor FIGO stage (*P* = .026). Multivariate survival analysis showed that FOXP4-AS1was an independent prognostic marker for overall survival (OS; hazard ratio [HR]: 0.638; 95% confidence interval [CI]:0.467–0.871; *P* = .001) and disease-specific survival (DSS; HR: 0.649; CI: 0.476–0.885; *P* = .006). GSEA showed that High FOXP4-AS1 expression may active programmed cell death 1 (PD-1) signaling, the cytotoxic T lymphocyte-associated antigen-4 (CTLA4) pathway, the B cell receptor signaling pathway, apoptosis, fibroblast growth factor receptor (FGFR) signaling, and the Janus-activated kinase signal transducers and activators of transcription (JAK-STAT) signaling pathway. FOXP4-AS1 expression was negatively correlated with markers of immune cells, including aDC, cytotoxic cells and neutrophils.

**Conclusion:**

High FOXP4-AS1 expression has the potential to be a prognostic molecular marker of favorable survival in OVs.

## 1 Introduction

Ovarian cancer is a common and lethal malignancy in women and is the most common cause of gynecologic cancer deaths.^[1]^ The most common histological subtype of ovarian cancer is ovarian serous carcinoma. Most patients with ovarian cancer are diagnosed in advanced stages because of the lack of specific symptoms and the absence of effective early diagnostic methods, which leads to a poor prognosis.^[2–4]^ In recent years, common serum biomarkers that have been used to monitor ovarian cancer progression and prognosis have included CA125 and HE4, which are also commonly used to detect ovarian cancer recurrence after surgery or chemotherapy.^[5,6]^ However, these biomarkers lack both specificity and sensitivity in predicting cancer prognosis. Consequently, the development of more sensitive and specific biomarkers for the early diagnosis of ovarian cancer is urgently needed.

Long non-coding RNAs (lncRNAs) are commonly defined as non-protein coding transcripts longer than 200 nucleotides that do not encode proteins but are important for development, differentiation, or metabolism.^[7–9]^ In tumors, lncRNAs can act as oncogenes or tumor suppressors, which influence the development and progression of cancer by regulating proliferation, apoptosis, autophagy, metastasis, self-renewal, and survival via epigenetic, transcriptional, and post-transcriptional regulatory mechanisms.^[10–12]^ Furthermore, aberrant expression of lncRNAs can be detected in circulating cancer cells and in the serum and urine of cancer patients, which suggests that lncRNAs may serve as effective biomarkers for cancer detection and prognosis.^[13,14]^

Li et al reported for the first time that a novel lncRNA, Forkhead box P4 antisense RNA 1 (FOXP4-AS1), exhibited upregulated expression in patients with colorectal cancer at late tumor stages and was associated with poorer overall survival.^[15]^ Subsequently, upregulation of the FOXP4-AS1 expression was identified in osteosarcoma,^[16]^ prostate cancer,^[17]^ hepatocellular carcinoma,^[18]^ gastric cancer,^[19]^ cervical cancer,^[20]^ esophageal squamous cell carcinoma,^[21]^ and pancreatic ductal adenocarcinoma,^[22]^ and such upregulation was also indicative of poor prognosis. However, associations between FOXP4-AS1 and the progression, metastasis, or clinical features of ovarian cancer patients have rarely been reported.

FOXP4-AS1 has been demonstrated to participate in the development and progression of osteosarcoma^[16]^ and gastric cancer^[19]^ by regulating tumor suppressor gene LATS1 via binding to LSD1 and EZH2. FOXP4-AS1 lncRNA promotes cervical cancer cell proliferation, migration, and invasion through sponging miR-136–5p to regulate CBX4 expression.^[20]^ Moreover, functional assays revealed that knockdown of FOXP4-AS1 efficiently suppressed esophageal squamous cell carcinoma cell proliferation and induced apoptosis.^[21]^ Taken together, these findings suggest that FOXP4-AS1 may not only act as a potential therapeutic target, but also serve as a novel predictive biomarker of progression and prognosis of cancer.

Hence, the objective of the present study was to investigate the prognostic value of FOXP4-AS1 expression in human ovarian serous cystadenocarcinoma (OVs) based on data obtained from The Cancer Genome Atlas (TCGA). To gain further insight into the biological pathways involved in OVs pathogenesis related to the FOXP4-AS1 regulatory network, Gene Set Enrichment Analysis (GSEA) was performed. Herein, we demonstrate that high expression of FOXP4-AS1 correlates with the favorable survival in OVs. GSEA revealed that several pathways and biological processes were differentially enriched in FOXP4-AS1-related OVs, including programmed cell death 1 (PD-1) signaling, the cytotoxic T lymphocyte-associated antigen-4 (CTLA4) pathway, the B cell receptor signaling pathway, apoptosis, fibroblast growth factor receptor (FGFR) signaling, and the JAK-STAT signaling pathway. In addition, we used single sample GSEA (ssGSEA) analysis to investigate the correlation between FOXP4-AS1 and markers of tumor infiltration by immune cells. Our results illustrate the significance of FOXP4-AS1 in OVs and explore the potential mechanism of FOXP4-AS1 in regulating the progression of OVs. Furthermore, we demonstrate the potential for FOXP4-AS1 as a biomarker for OVs prognosis.

## 2 Materials and methods

### 2.1 Editorial policies and ethical considerations

This article does not contain any experiments involving human participants or animals.

### 2.2 FOXP4-AS1 sequencing data acquisition and processing

We followed the methods of Ma et al.^[23]^ Clinical information and gene expression data from STAD projects were collected from TCGA (https://tcga-data.nci.nih.gov/tcga/).^[24]^ The inclusion criteria for the database were the following:

1. including pathological diagnosis;
2. no neoadjuvant therapy, including chemotherapy, radiotherapy, or immunotherapy;
3. complete survival information.

The exclusion criteria were normal STAD samples and an overall survival <30 days. The corresponding prognosis information also referred to the University of California Santa Cruz (UCSC) Xena (https://xenabrowser.net/heatmap/).^[25]^ Gene-level transcriptome profiling (*RNA-seq*) data in level 3 high-throughput sequencing fragments per kilobase per million (HTSeq-FPKM) format were converted to transcripts per million reads (TPM) format for subsequent analysis. The data are summarized in Table 1.

**Table 1:** TCGA ovarian serous cystadenocarcinoma patient characteristics.

Characteristic		Overall
n		376
OS event (%)	Alive	146 (38.8)
	Dead	230 (61.2)
FIGO stage (%)	Stage I	1 (0.3)
	Stage II	22 (5.9)
	Stage III	293 (78.6)
	Stage IV	57 (15.3)
Histologic grade (%)	G1	1 (0.3)
	G2	42 (11.5)
	G3	322 (88.0)
	G4	1 (0.3)
Tumor status (%)	Tumor free	71 (21.3)
	With tumor	262 (78.7)
Primary therapy outcome (%)	PD	27 (8.9)
	SD	22 (7.2)
	PR	43 (14.1)
	CR	213 (69.8)
Age (%)	<60	198 (52.7)
	≥60	178 (47.3)
Residual tumor (%)	NRD	66 (19.8)
	RD	267 (80.2)
Lymphatic invasion (%)	NO	48 (32.4)
	YES	100 (67.6)
Venous invasion (%)	NO	40 (38.8)
	YES	63 (61.2)
Anatomic subdivision (%)	Unilateral	101 (28.5)
	Bilateral	253 (71.5)

CR = complete remission, NRD = no residual disease, OS = overall survival, PD = progressive Disease, PR = partial remission, RD = residual disease, SD = stable disease, TCGA = The Cancer Genome Atlas.

### 2.3 Analysis of gene set enrichment

GSEA (http://software.broadinstitute.org/gsea/index.jsp) is a computational method that determines whether a set of genes defined a priori show statistically significant and concordant differences between two biological states.^[26]^ Through research based on the correlation with FOXP4-AS1 expression, GSEA was the first to rate all the genes in an ordered list to elucidate the significant survival difference, which is observed between groups expressing high and low FOXP4-AS1 levels. In addition, setting permutations were performed 1000 times for each analysis. The expression profiles of FOXP4-AS1 were used as phenotypic labels. Nominal *P*-values and normalized enrichment scores (NES) were used to rank the pathways with FOXP4-AS1 enrichment in each phenotype.

### 2.4 Immune infiltration analysis

The marker genes for immune cell types were referred from a previous study by Bindea et al.^[27]^ The infiltration levels of the immune cell types were quantified by a ssGSEA using the GSVA pacakage39 in the R software. Spearman's correlation was used to analyze the correlation between expression of the FOXP4-AS1 gene and that of the 24 immune cell markers. The correlation between the expression of FOXP4-AS1 and immune cell markers is indicated by the following values: 0.00 to 0.05, very weak; 0.06 to 0.10, weak; 0.11 to 0.15, moderate; and >0.15, strong. For statistical analyses, *P*-values of <.05 were considered to indicate statistical significance.

### 2.5 Statistical analysis

The R software (version 3.6.2; http://www.Rproject.org) was used for all the statistical analyses. Wilcoxon rank sum test was used to compare the expression of FOXP4-AS1 in OVs and normal ovarian tissue. Logistic regression was used to analyze the relationship between clinicopathologic features and FOXP4-AS1. Kaplan–Meier method was used to generate survival curves, and Cox regression was used to analyze the relationship between FOXP4-AS1 and survival rate. The individual hazard ratio (HR) of the operating system was estimated by univariate Cox proportional hazards regression. Elements with significance levels of *P* < .1 in univariate analysis were included in multivariate Cox analysis. HR and 95% confidence intervals (CI) were measured to estimate associations of individual factors. The *P*-values of all results were bilateral with values <.05 indicating significance.

## 3 Results

### 3.1 Association between FOXP4-AS1 expression and clinicopathologic variables

As shown in Table 1, 376 OVs were divided into two groups by age. The percentage of patients younger than 60 years was 52.7, and that of those older than 60 years was 47.3. Of those patients who were followed, 146 (38.8%) survived, and 230 (61.2%) died. Most patients were FIGO stage III (293 cases; 78.6%) or stage IV (57 cases; 15.3%). G3 histologic grade characterized 322 (88%) patients. Among subjects in this study, 71 (21.3%) were tumor free and 262 (78.7%) cases had a tumor. Primary therapy outcomes included 8.9% progressive disease (PD), 7.2% stable disease (SD), 14.1% partial remission (PR), and 69.8% complete remission (CR). Among 333 patients who were assessed for residual disease, 267 (80.2%) had residual tumor, while 66 (19.8%) had no residual disease. Out of 148 cases that were assessed, 100 (67.6%) had lymphatic invasion and 63 (61.2%) of 103 cases had venous invasion. Anatomic subdivision was bilateral in 253 of 354 (71.5%) cases.

**Figure 1:**
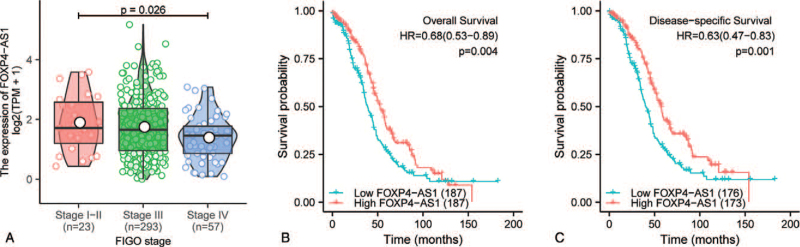
Clinical correlation and prognosis analysis of FOXP4-AS1 in ovarian serous cystadenocarcinoma patients in the TCGA cohort. Association of FOXP4-AS1 expression with FIGO stage (A), statistical analysis method: Kruskal–Wallis rank sum test. Relationship between FOXP4-AS1 expression and overall survival (B). Relationship between FOXP4-AS1 expression and disease-specific survival (C). TCGA = The Cancer Genome Atlas.

**Figure 2:**
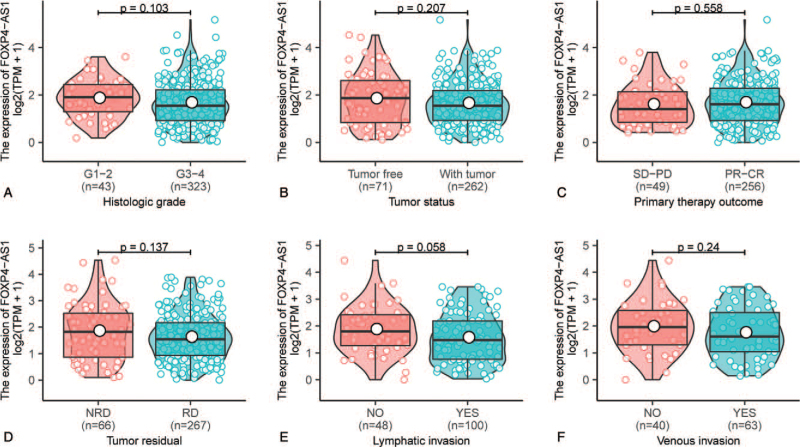
Relationship between FOXP4-AS1 expression and clinical characteristics of ovarian serous cystadenocarcinoma patients. Association of FOXP4-AS1 expression with clinicopathologic characteristics including histologic grade (A), tumor status (B), primary therapy outcome (C), residual tumor (D), lymphatic invasion (E), and venous invasion (F) in patients with ovarian serous cystadenocarcinoma in The Cancer Genome Atlas cohort. CR = complete remission, NRD = no residual disease, PD = progressive disease, PR = partial remission, RD = residual disease, SD = stable disease. Analysis between the two groups: Wilcoxon rank sum test.

As shown in Figure 1A and Figure 2, High FOXP4-AS1 expression correlated significantly with clinical stage (*P* = .026, Fig. 1A). However, other clinicopathologic characteristics including histologic grade (*P* = .103, Fig. 2A), tumor status (*P* = .207, Fig. 2B), primary therapy outcome (*P* = .558, Fig. 2C), residual tumor (*P* = .137, Fig. 2D), lymphatic invasion (*P* = .058, Fig. 2E), and venous invasion (*P* = .24, Fig. 2F), there were not significant differences.

### 3.2 Univariate and multivariate analyses of survival

Kaplan–Meier survival analysis showed that high FOXP4-AS1 expression correlated significantly with favorable overall survival (OS) (HR: 0.68; 95% CI: 0.53–0.89; *P* = .004, Fig. 1B) and disease-specific survival (DSS) (HR: 0.63; 95% CI: 0.47–0.83; *P* = .001, Fig. 1C). Univariate and multivariate Cox regression analyses were performed to determine whether FOXP4-AS1 expression was an independent prognostic indicator in OVs. Univariate analysis revealed that high FOXP4-AS1 expression associated significantly with favorable OS (HR: 0.685; 95% CI: 0.528–0.889; *P* = .004). Other clinicopathologic variables associated with favorable OS included primary therapy outcome of PR-CR, <60 years of age and no residual disease. In multivariate analysis, FOXP4-AS1 remained independently associated with OS (HR: 0.638; 95% CI: 0.467–0.871; *P* = .005) (Table 2). In addition, univariate Cox regression analysis of DSS was performed, indicating that high FOXP4-AS1 expression associated significantly with favorable DSS (HR: 0.626; 95% CI: 0.472–0.829; *P* = .001). Other clinicopathologic variables associated with favorable DSS included primary therapy outcome of PR-CR and no residual disease. In multivariate analysis, FOXP4-AS1 remained independently associated with DSS (HR: 0.649; 95% CI: 0.476–0.885; *P* = .006) (Table 3).

**Table 2:** Univariate and multivariate Cox proportional hazards analysis of FOXP4-AS1 expression and OS for patients with OVs in the validation cohort.

	Univariate analysis		Multivariate analysis	
Characteristic	Hazard ratio (95% CI)	*P*	Hazard ratio (95% CI)	*P*
FIGO stage (Stage I–II vs. Stage III–IV)	2.085 (0.925–4.699)	.076	2.817 (0.691–11.485)	.149
Histologic grade (G1–2 vs G3–4)	1.194 (0.797–1.789)	.389		
Primary therapy outcome (SD-PD vs PR-CR)	0.306 (0.207–0.451)	**<.001**	0.313 (0.206–0.474)	**<.001**
Age (<60 vs >=60)	1.329 (1.025–1.722)	**.032**	1.204 (0.881–1.645)	.243
Tumor residual (NRD vs RD)	2.302 (1.479–3.583)	**<.001**	2.062 (1.243–3.419)	**.005**
Lymphatic invasion (NO vs YES)	1.422 (0.839–2.411)	.191		
Venous invasion (NO vs YES)	0.905 (0.487–1.683)	.753		
Anatomic subdivision (unilateral vs bilateral)	1.041 (0.768–1.41)	.798		
FOXP4-AS1 (low vs high)	0.685 (0.528–0.889)	**.004**	0.638 (0.467–0.871)	**.005**

CI = confidence interval, CR = complete remission, NRD = no residual disease, OS = overall survival, OVs = ovarian serous cystadenocarcinoma, PD = progressive disease, PR = partial remission, RD = residual disease, SD = stable disease.

**Table 3:** Univariate and multivariate Cox proportional hazards analysis of FOXP4-AS1 expression and DSS for patients with OVs in the validation cohort.

	Univariate analysis		Multivariate analysis	
Characteristic	Hazard ratio (95% CI)	*P*	Hazard ratio (95% CI)	*P*
FIGO stage (stage I–II vs stage III–IV)	2.244 (0.922–5.462)	.075	2.789 (0.684–11.379)	.153
Histologic grade (G1–2 vs G3–4)	1.313 (0.833–2.07)	.24		
Primary therapy outcome (SD-PD vs PR-CR)	0.299 (0.201–0.443)	**<.001**	0.317 (0.21–0.48)	**<.001**
Age (<60 vs ≥60)	1.248 (0.944–1.65)	.12		
Tumor residual (NRD vs RD)	2.559 (1.572–4.166)	**<.001**	2.066 (1.246–3.427)	**.005**
Lymphatic invasion (NO vs YES)	1.407 (0.816–2.425)	.219		
Venous invasion (NO vs YES)	0.846 (0.45–1.591)	.604		
Anatomic subdivision (unilateral vs bilateral)	1.034 (0.747–1.431)	.841		
FOXP4-AS1 (low vs high)	0.626 (0.472–0.829)	**.001**	0.649 (0.476–0.885)	**.006**

CI = confidence interval, CR = residual disease, DSS = disease-specific survival, NRD = no residual disease, OVs = ovarian serous cystadenocarcinoma, PD = progressive disease, PR = partial remission, RD = residual disease, SD = stable disease.

### 3.3 Gene sets enriched in FOXP4-AS1 expression phenotype

**Figure 3:**
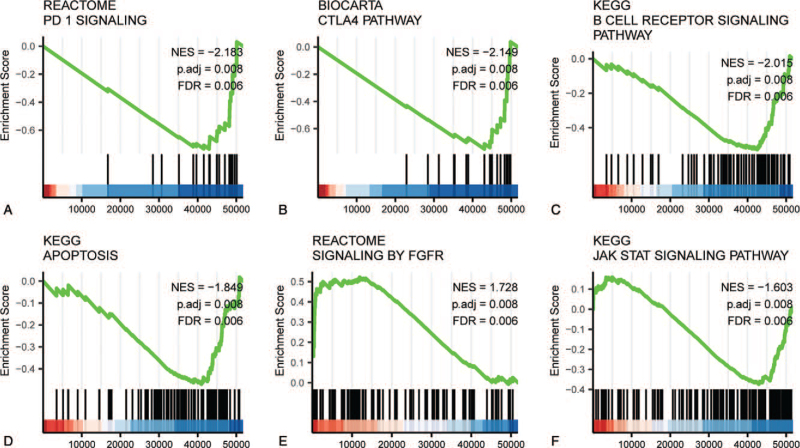
Enrichment plots from gene set enrichment analysis (GSEA). GSEA results showing differential enrichment of PD-1 signaling (A), CTLA4 pathway (B), B cell receptor signaling pathway (C), apoptosis (D), signaling by FGFR (E), JAK-STAT signaling pathway (F) in FOXP4-AS1-related ovarian cancer. ES = enrichment score, FDR = false discovery rate, NES = normalized ES, *p*-adjust = adjusted *P*-value.

We used GSEA analysis of a TCGA expression dataset to identify diverging functional and biological pathways between low and high FOXP4-AS1 expression groups. Based on the standardized enrichment fraction (NES) we chose the enrichment signaling pathways (Fig. 3 and Table 4) that were most significantly associated with FOXP4-AS1 expression. In Figure 3, the results of GSEA show that the group with high expression of FOXP4-AS1 was mainly enriched in programed cell death protein 1 (PD-1) signaling (Fig. 3A), the CTLA4 pathway (Fig. 3B), the B cell receiver signaling pathway (Fig. 3C), apoptosis (Fig. 3D), FGFR signaling (Fig. 3E), and the JAK-STAT signaling pathway (Fig. 3F).

**Table 4:** Gene sets enriched in phenotype.

Gene set name	Set size	Enrichment score	NES	NOM *P*	*p*-Adjust	FDR *q*
High expression						
REACTOME_PD_1_SIGNALING	23	<0.001	<0.001	<.001	0.008	0.006
BIOCARTA_CTLA4_PATHWAY	20	<0.001	<0.001	<.001	0.008	0.006
KEGG_B_CELL_RECEPTOR_SIGNALING_PATHWAY	75	<0.001	<0.001	<.001	0.008	0.006
KEGG_APOPTOSIS	87	<0.001	<0.001	<.001	0.008	0.006
REACTOME_SIGNALING_BY_FGFR	86	0.522	1.728	<.001	0.008	0.006
KEGG_JAK_STAT_SIGNALING_PATHWAY	154	<0.001	<0.001	.001	0.008	0.006
Low expression						
REACTOME_INTERLEUKIN_21_SIGNALING	10	−0.852	−1.99	<.001	0.039	0.031
BIOCARTA_IL5_PATHWAY	11	−0.8254	−2.015	<.001	0.039	0.031
REACTOME_INTERLEUKIN_2_SIGNALING	12	−0.76774	−1.90938	<.001	0.039	0.031
BIOCARTA_MTA3_PATHWAY	18	−0.67389	−1.85959	<.001	0.039	0.031
BIOCARTA_CD40_PATHWAY	15	−0.71742	−1.86772	<.001	0.039	0.031
BIOCARTA_IL17_PATHWAY	15	−0.7526	−1.95831	<.001	0.039	0.031

FDR *q*-values, which is the probability estimate of possible false positive results for NES; Differences in gene sets with NOM *P*-value <.05 and FDR *q*-values <0.05 were considered statistically significant. FDR = false discovery rate, NES = normalized enrichment score, NOM = nominal.

### 3.4 Correlation analysis between FOXP4-AS1 expression and immune infiltration

**Figure 4:**
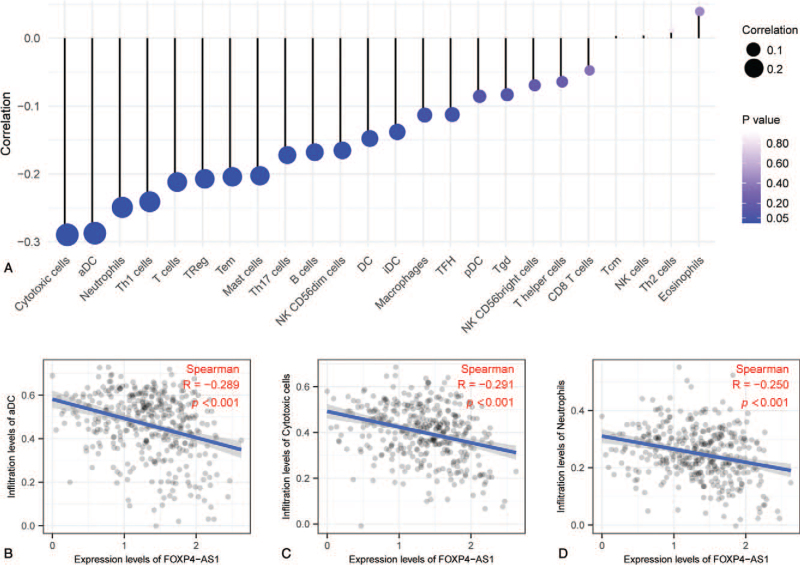
Correlation analysis between FOXP4-AS1 and related immune cell markers in ovarian serous cystadenocarcinoma. Correlation analysis between FOXP4-AS1 expression and immune cells (A); Correlation analysis between FOXP4-AS1 expression and levels of DC cell infiltration markers (B) and those of cytotoxic cells (C) and neutrophils (D).

We then analyzed correlation between the expression level of FOXP4-AS1 and immune cell enrichment level by Spearman's correlation and found that FOXP4-AS1 expression was negatively correlated with markers of immune cells, including cytotoxic cells, activated dendritic cells (aDC), neutrophils, TH1 cells, T cells, TRegs, Tmems, Mast cells, and TH17 cells (Fig. 4A). Further investigation showed that FOXP4-AS1 expression was significantly correlated with the level of markers of infiltration of aDC (*P* < .001, *R* = −0.289) (Fig. 4B), cytotoxic cells (*P* < .001, *R* = −0.291) (Fig. 4C), and neutrophils (*P* < .001, *R* = −0.250) (Fig. 4D).

## 4 Discussion

Recent studies have shown that FOXP4-AS1 lncRNA plays an important role in various cancer types,^[15–22]^ and might act to promote cancer cell proliferation and invasion. FOXP4-AS1 lncRNA has been reported to be involved in the proliferation and invasion of osteosarcoma and gastric cancer cells by regulating the expression of LSD1. LSD1 was reported to be involved in the development of ovarian cancer.^[28]^ However, the association between FOXP4-AS1 lncRNA and ovarian cancer has rarely been reported.^[29]^ Therefore, our aim in this study was to elucidate the expression of FOXP4-AS1 in ovarian cancer tissues and the potential therapeutic and prognostic value of this lncRNA. Our results show that high FOXP4-AS1 expression in OVs was associated with favorable prognosis. Additionally, we demonstrate that FOXP4-AS1 expression levels in OVs were correlated with markers for different types of immune-infiltrating cells. Thus, the current evidence supports a potential role of FOXP4-AS1 in OVs immunity and as a prognostic marker of this malignancy or a potential diagnostic.

Based on the database, high FOXP4-AS1 expression was significantly correlated with tumor FIGO stage (*P* = .026). There were no significant correlations between high FOXP4-AS1 expression and histologic grade, tumor status, primary therapy outcomes, residual tumors, lymphatic and venous infiltrations. In addition, we used GSEA to examine the function of FOXP4-AS1 in OVs tissues. In tissues with high expression of FOXP4-AS1, pathways including PD-1 signaling, the CTLA4 pathway, the B cell receiver signaling pathway, apoptosis, FGFR signaling, and the JAK-STAT signaling pathway were found be differentially enriched compared to tissues with low FOXP4-AS1 expression. These pathways have been reported to associate with OVs.^[30–32]^ Moreover, our univariate and multivariate regression analyses revealed that high levels of FOXP4-AS1 expression were correlated with favorable OS and DSS. Together, our findings suggest that FOXP4-AS1 may be a prognostic biomarker for OVs.

In OVs, the current study reveals that FOXP4-AS1 expression is related to markers for a variety of infiltrating immune cells, including aDC, cytotoxic cells, and neutrophils. Therefore, we posit that the role of FOXP4-AS1 as a tumor suppressor gene may be related to these immune cells in OVs. Dendritic cells (DCs) are professional antigen presenting cells that endocytose exogenous antigens^[33]^ and have the ability to regulate the type of T cell-mediated immune response.^[34]^ In tumor immunity, DCs initially take up tumor-associated antigens in tumor tissue and presumably migrate to regional lymph nodes to generate tumor-specific immunity.^[35]^ In detailed studies of CD4(+) CD25(+) FOXP3(+) T-reg cells in 104 individuals with ovarian carcinoma, Curiel et al found that human tumor T-reg cells suppress tumor-specific T cell immunity and contribute to the growth of human tumors in vivo. They also found that tumor T-reg cells are associated with a high death hazard and reduced survival.^[36]^ Our results indicate that FOXP4-AS1 has a strong negative correlation with DCs and T-reg markers. Studies have demonstrated that tumor infiltrating lymphocytes (TILs) and inflammation correlate with good and bad prognoses, respectively.^[37,38]^ TIL is a term that refers to lymphocytes that extravasate from blood vessels and access the tumor. Typical TILs include T cells, macrophages, NK cells, and DCs.^[39]^ Tumor cells can suppress the immune response of TILs in various ways. In contrast, tumor cells can directly inhibit antitumor immune cells, but on the other hand, they can activate the cell subsets which play the role of immune suppression so as to achieve the goal of immune escape. For instance, the combination of programmed death receptor-ligand 1(PD-L1) expressed by tumor cells and PD-1 expressed by CD8+ toxic T cells can promote the inactivation or apoptosis of CD8+ toxic T cells, thus weakening the host's antitumor immune response.^[39]^ CD8+ toxic T cells are cytotoxic lymphocytes that damage targeted cells via the production of enzymes such as granzyme-B and perforin.^[38]^ Our results imply that FOXP4-AS1 has a strong negative correlation with the infiltration of cytotoxic cells. Systemic inflammation has been consistently associated with poor clinical outcomes. Specifically, the prognostic value of elevated neutrophil and lymphocyte counts have been conclusively demonstrated.^[30]^ In our study, the expression of neutrophil markers decreased as the expression of FOXP4-AS1 increased. Reduced inflammation is associated with better outcomes in ovarian cancer as demonstrated by previous studies.^[30,37]^ Our findings suggest that FOXP4-AS1 plays an important role in the regulation of immune cell infiltration and inflammatory response in OVs.

We provide evidence that high FOXP4-AS1 expression plays a tumor suppressive role in OVs by participating in biological processes and pathways including PD-1 signaling, the CTLA4 pathway, the B cell receptor signaling pathway, apoptosis, signaling by FGFR, and the JAK-STAT signaling pathway. It is well known that immunoregulatory proteins PD-1 and CTLA4 attenuate the immune system in tumors. Studies have shown that blocking PD-1 and CTLA4 can modulate T-reg functions and enhance antitumor responses.^[31]^ Nielsen et al demonstrated that CD20+ B cells co-localized with activated CD8+ TILs expressing antigen presentation markers and correlated with increased patient survival in ovarian cancer compared to patients with CD8+ TILs alone.^[40]^ The FGF/FGFR family consists of 19 FGFs and four FGFRs that can interact with the PI3K/AKT pathway and subsequently inhibit ovarian cancer growth.^[41]^ In addition, FGFRs recruit stromal cells, which are essential participants in the growth and motility of ovarian cancer cells. Cai et al demonstrated that simultaneous inhibition of FGFR and mTOR activity contributed to anti-proliferative effects and tumor regression in ovarian cancer.^[32]^ The JAK/STAT signaling pathway plays a vital role not only in the transformation of stationary epithelial cells to invasive and migratory cells but also in the maintenance of stem cell self-renewal.^[42]^ Therefore, we speculate that FOXP4-AS1 plays an important role as an anticancer gene inhibiting the transformation of ovarian epithelial cells into invasive and migratory cells through this pathway.

Although our current research methods have greatly improved our understanding of the relationship between FOXP4-AS1 and OVs, some limitations remain. First, in order to fully elucidate the specific role of FOXP4-AS1 in the development of OVs, it is necessary to include multiple clinical parameters of patients receiving ovarian cancer treatment. Secondly, there was an imbalance in the number of healthy control subjects and the number of cancer patients in the current study. A larger control sample in future studies will increase the resolution and power of statistical analyses. Thirdly, sample size prevented the analysis of the effect of ethnicity and geography on FOXP4-AS1 expression in OVs. Retrospective studies have limitations due to a lack of specific information. Therefore, future prospective studies are justified. Since this study mainly relied on the RNA sequencing results from TCGA database, we were unable to directly delineate the mechanism of FOXP4-AS1 function in OVs development. Therefore, the direct mechanism of OVs requires further evaluation.

In conclusion, FOXP4-AS1 expression may be a molecular prognostic marker of poor survival in OVs. Moreover, PD-1 signaling, the CTLA4 pathway, the B cell receptor signaling pathway, apoptosis, FGFR signaling, and the JAK-STAT signaling pathway may be regulated by FOXP4-AS1 in OVs. To identify the biological role of FOXP4-AS1 in OVs, further experimental validation is justified.

## Acknowledgments

The authors would like to thank Dr Huimei Wang for the English language review and statistical Analysis. Dr Huimei Wang is a statistic professional at helixlife, a well-known research and teaching platform for clinicians in China (https://www.helixlife.cn/).

## Author contributions

CL and HL made substantial contributions to the conception and design of the present study. AW and YSM performed data acquisition, data analysis, and interpretation. HL drafted the manuscript and critically revised it for important intellectual content. All authors read and approved the final manuscript.

**Conceptualization:** Hui Liu.

**Data curation:** Cheng Liao, Ao Wang, Yushan Ma.

**Project administration:** Hui Liu.

**Software:** Cheng Liao, Ao Wang, Yushan Ma.

**Writing – original draft:** Cheng Liao.

**Writing – review & editing:** Hui Liu.
